# Binding and functional structure-activity similarities of 4-substituted 2,5-dimethoxyphenyl isopropylamine analogues at 5-HT_2A_ and 5-HT_2B_ serotonin receptors

**DOI:** 10.3389/fphar.2023.1101290

**Published:** 2023-01-24

**Authors:** Prithvi Hemanth, Pallavi Nistala, Vy T. Nguyen, Jose M. Eltit, Richard A. Glennon, Małgorzata Dukat

**Affiliations:** ^1^ Department of Medicinal Chemistry, School of Pharmacy, Virginia Commonwealth University, Richmond, VA, United States; ^2^ Department of Physiology and Biophysics, School of Medicine, Virginia Commonwealth University, Richmond, VA, United States

**Keywords:** 5-HT_2A_, 5-HT_2B_, Ca^2+^ mobilization assay, agonist, antagonist, QSAR, psychedelic agents, valvulopathy

## Abstract

Certain 4-substituted analogs of 1-(2,5-dimethoxyphenyl)isopropylamine (2,5-DMA) are psychoactive classical hallucinogens or serotonergic psychedelic agents that function as human 5-HT_2A_ (h5-HT_2A_) serotonin receptor agonists. Activation of a related receptor population, h5-HT_2B_ receptors, has been demonstrated to result in adverse effects including cardiac valvulopathy. We previously published on the binding of several such agents at the two receptor subtypes. We hypothesized that, due to their structural similarity, the 5-HT_2A_ and 5-HT_2B_ receptor affinities of these agents might be related, and that QSAR studies might aid future studies. For a series of 13 compounds, it is demonstrated here that i) their published rat brain 5-HT_2_ receptor affinities are significantly correlated with their h5-HT_2A_ (r = 0.942) and h5-HT_2B_ (r = 0.916) affinities, ii) as with r5-HT_2_ receptor affinity, h5-HT_2A_ affinity is correlated with the lipophilicity of the 4-position substituent (r = 0.798), iii) that eight of the ten compounds examined in functional (Ca^+2^ mobilization in stable cell lines generated expressing the human 5-HT_2B_ receptor using the Flp-In T-REx system) assays acted as h5-HT_2B_ agonists (4-substituent = H, F, Br, I, OCH_2_CH_3_, NO_2_, *n*C_3_H_7_, *t*C_4_H_9_) and two (*n-*hexyl and benzyl) as antagonists, iv) h5-HT_2B_ affinity but not action was correlated with the lipophilicity of the 4-position substituent (r = 0.750; *n* = 10). The findings suggest that h5-HT_2B_ receptor affinity, and its relationship to substituent lipophilicity, might be approximated by rat and h5-HT_2A_ affinity but cannot be used as a predictor of h5-HT_2B_ agonist action of 2,5-DMA analogs. Furthermore, given that certain 2,5-DMA analogs are on the clandestine market, their potential to produce cardiac side effects following persistent or chronic use *via* activation of h5-HT_2B_ receptors should be considered.

## 1 Introduction

More than 35 years ago, and shortly following the discovery of rat brain 5-HT_2_ (now considered 5-HT_2A_) serotonin receptors, phenylisopropylamine analogues were identified as a novel 5-HT_2_ receptor chemotype (Shannon et al., 1984). In fact, some evidence for this was available from earlier studies using peripheral 5-HT receptor preparations (Glennon et al., 1978) (i.e., rat fundus 5-HT receptors; years later, initially termed 5-HT_2F_—“F” for rat “fundus”—receptors, these are now considered 5-HT_2B_ serotonin receptors (Kursar et al., 1992)). Certain methoxy-substituted phenylisopropylamines, particularly 2,5-dimethoxy analogs **1** with different R substituents at the aryl 4-position, displayed varying and nanomolar affinities for rat fundus and, later, rat brain 5-HT_2_ receptors (Seggel et al., 1990), and their affinities were correlated both with their discriminative stimulus properties in rats and their human hallucinogenic potencies (reviewed: Glennon 1996).

In an effort to understand how the 4-position R-substituents of **1** influence 5-HT_2_ receptor affinity, an extended series of analogs (*n* = 24) was prepared and a QSAR study conducted (Glennon and Seggel 1989; Seggel et al., 1990). Using rat brain frontal cortex homogenates with the 5-HT_2_ receptor antagonist [^3^H]ketanserin as radioligand, QSAR studies employing rat 5-HT_2_ binding data revealed, for these two dozen analogues of **1** with affinities spanning over a >10,000-fold range (i.e., *K*_i_ = 2.5 to 26,000 nM), that affinity was related to the lipophilicity (i.e., π value) and electron withdrawing character (i.e., σ_p_) of the 4-position substituent (Glennon and Seggel 1989). That is, increasing the lipophilicity and the electron withdrawing character of the 4-position R group seemed responsible for rat 5-HT_2_ receptor affinity. There was also a hint that the “overall size” of the 4-R substituent might play a role in binding (Glennon and Seggel 1989).

Follow-up binding studies were conducted on a sub-set of representative 2,5-DMA (**1a**) (i.e., **1**; R = H for 2**,**5-DMA) analogues from the rat brain homogenate studies with cloned human 5-HT_2A_ serotonin receptors using [^125^I]DOI, a 5-HT_2_ agonist radioligand (i.e., radioiodinated **1** where R = ^125^I) (Nelson et al., 1999); data for compounds common to the two investigations discussed here are shown in Table 1-A). Human 5-HT_2B_ receptor binding data were also reported in the same investigation. Neither functional nor QSAR studies were performed with the human data in the previous investigation.

**TABLE 1:** A: Published radioligand binding data (p*K*_i_ values) for a series of analogues **1** common to human 5-HT_2A_ vs. [^125^I]DOI, human 5-HT_2B_ vs. [^3^H]5-HT (Nelson et al., 1999) and rat 5-HT_2_ vs. [^3^H]ketanserin (Seggel et al., 1990) receptor studies, and B: agonist potencies (EC_50_ values) and maximal 5-HT-related effect (5-HT = 100%) at human 5-HT_2B_ (h5-HT_2B_) receptors as determined in this study.

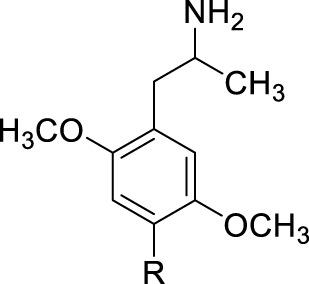							

A						B	
	R	Acronym	h5-HT_2A_ p*K*_i_	r5-HT_2_ p*K*_i_	h5-HT_2B_ p*K*_i_	h5-HT_2B_ pEC_50_ ± SEM (EC_50_)^a^	Maximal 5-HT effect^b^
**1a**	H	2,5-DMA	6.68	5.28	5.98	5.47 ± 0.11 (3,386 nM)	93%
**1b**	F	DOF	7.38	5.96	6.64	6.36 ± 0.12 (439 nM)	82%
**1c**	Cl	DOC	8.85	6.66	7.50	NA	-
**1d**	Br	DOB	9.22	7.39	7.53	8.06 ± 0.23 (8.7 nM)	70%
**1e**	I	DOI	9.15	7.72	7.70	7.43 ± 0.17 (39 nM)	71%
**1f**	OCH_3_	2,4,5-TMA	7.24	5.90	6.51	NA	-
**1g**	OCH_2_CH_3_	MEM	7.14	5.66	6.12	6.25 ± 0.24 (557 nM)	70%
**1h**	NO_2_	DON	8.26	6.52	6.78	7.05 ± 0.13 (89 nM)	77%
**1i**	CN	DOCN	7.34	5.62	6.11	NA	-
**1j**	nPr	DOPR	9.05	7.16	7.26	7.26 ± 0.19 (30 nM)	75%
**1k**	nHex	DOHX	10.00	8.60	7.52	<5.0^c^ (>10,000 nM)	0%
**1l**	*t*Bu	DOTB	8.43	7.72	7.61	7.43 ± 0.20 (37 nM)	69%
**1m**	Benzyl	DOBz	9.40	8.15	7.64	<5.0^c^ (>10,000 nM)	0%

^a^ NA, Not assayed. ^b^Maximal effect relative to 5-HT (pEC_50_ = 8.85 ± 0.14; EC_50_ = 1.42 nM) = 100%.

^c^ No agonist effect up to this concentration.

The psychoactive properties of classical hallucinogens (including certain phenylisopropylamines **1**) appear to involve their agonist action at 5-HT_2A_ serotonin receptors (reviewed: Glennon 1996), whereas activation of 5-HT_2B_ receptors can lead to several serious cardiovascular problems (e.g. vavulopathy) (Rothman et al., 2000; Setola et al., 2005; Roth 2007; Elangbam 2010). The goals of the current study were: i) to determine if the SAR of analogues **1** (Table 1) at human 5-HT_2A_ receptors are related to their SAR at rat 5-HT_2_ receptors, ii) to affirm (or counter) previous rat 5-HT_2_ binding QSAR findings for common analogues **1** by examining their human 5-HT_2A_ receptor affinities, iii) to investigate the possibility that human 5-HT_2A_ SAR and QSAR results might inform human 5-HT_2B_ receptor action, and iv) to determine if 5-HT_2B_ receptor affinity and binding QSAR findings are a reliable predictor of 5-HT_2B_ agonist action. These results could have substantial translational or clinical ramifications on the abuse of psychoactive 5-HT_2_ receptor agonists regarding their potential for producing adverse cardiac events.

## 2 Materials and methods

### 2.1 Materials

Compounds examined in the functional assays were available as their hydrochloride salts from earlier synthetic studies conducted in our laboratory.

### 2.2 5-HT_2B_ receptor functional activity using Ca^2+^ mobilization assay

A stable cell line was generated expressing the human 5-HT_2B_ receptor using the Flp-In T-REx system (Thermo Fisher) (Younkin et al., 2016; Steele et al., 2021). Briefly, 5-HT_2B_ receptor coding plasmid was obtained from cDNA Resource Center (cat # HTR02B0000). The 5-HT_2B_ receptor cDNA was subcloned into the pcDNA/FRT/TO expression plasmid, and was co-transfected with pOG44 plasmid (coding the Flp recombinase) in Flp-In T-REx 293 cells. Cells were selected using 100 μg/mL of hygromycin and resistant cells were expanded and stored in liquid N_2_ for later use. For experimentation, stable cell lines were plated in Matrigel-coated 96-well imaging plates in Dulbecco’s Modified Eagle Medium (DMEM) containing 10% fetal bovine serum (FBS) and 5% penicillin/streptomycin, and the medium was supplemented with doxycycline (1 μg/mL) to upregulate the expression of the receptor 3 days before the experiment. Then, cells were loaded with Fura 2-AM for 30 min in Ca^2+^ imaging solution (IS) consistent of 130 NaCl, 4 KCl, 2 CaCl_2_, 1 MgCl_2_, 10 HEPES, 10 glucose (in mM, pH adjusted to 7.4). Ca^2+^ measurements were performed in IS media at room temperature under constant perfusion using the equipment previously described in Ruchala et al. (2021). Fura2 was visualized using epifluorescence microscopy at two excitation wavelengths (340 nm and 380 nm) and a common single emission (510/40 nm). Ratio images were recorded, and the effect of each compound was tested at a single concertation per well and compared to the effect of 1 µM 5-HT. Antagonists were tested by inhibiting 10 nM 5-HT Ca^2+^ signal, preincubating them for 45 s in combination with 5-HT. Three wells containing cells were analyzed for each concentration per experiment day. Two experiments were conducted for each drug testing at least 6 wells per experimental point in total. Data are depicted as mean ± SD. All data were processed used Fiji software by ImageJ2 which allowed manual selection of cells and measurement of fluorescence. Logarithmic concentration-response curves were generated using GraphPad Prism 8. Curves were generated with a Hill slope of 1.0 to allow easier comparison of potencies of the various compounds analyzed.

### 2.3 Plotting

Data were plotted and correlations and QSAR regression analysis and multiple linear regression analysis were conducted using GraphPad Prism 9.03.

## 3 Results

### 3.1 Relationship between rat and human 5-HT_2_ receptor binding

For 13 compounds common to the rat and human 5-HT_2A_ receptor studies (Table 1-A), affinities were higher at the human 5-HT_2A_ than at rat 5-HT_2_ receptors, but there was a significant correlation between their receptor affinities (r = 0.942; Supplementary Figure S1; SI) despite differences in methods, species, and radioligands employed. Likewise, there was a significant correlation (r = 0.916) between h5-HT_2B_ and rat 5-HT_2_ receptor affinities (Table 1-A) for these same 13 compounds (Supplementary Figure S2; SI).

### 3.2 5-HT_2B_ receptor affinity and functional activity

**FIGURE 1:**
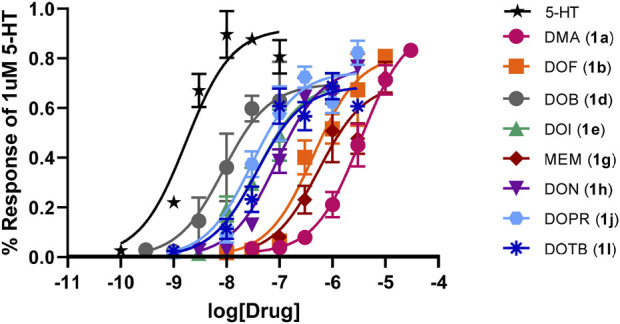
Concentration-response curves for eight agonists (Table 1) normalized to 1 µM of the endogenous ligand (5-HT) 5-HT_2B_ response.

**FIGURE 2:**
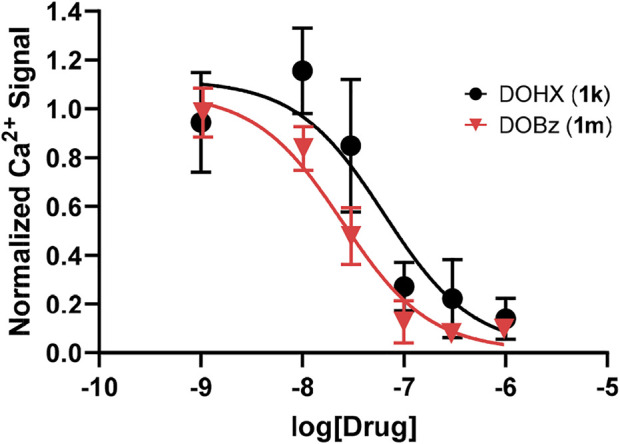
5-HT_2B_ receptor antagonist data with **1k** (DOHX) pIC_50_ = 7.2 ± 0.18 (63.6 nM) and **1m** (DOBz) pIC_50_ = 7.61 ± 0.08 (24.6 nM) in the presence of 10 nM of 5-HT.

The **1** analogs bind at 5-HT_2B_ receptors (Table 1-A). Ten of the compounds in Table 1-B (**1a**, **1b**, **1d**, **1e**, **1g**, **1h**, **1j**, **1k**, **1l**, **1m**) were examined in comparison with 5-HT for their functional activity at human 5-HT_2B_ receptors expressed in HEK 293 (Flp-In T-REx) cells. The compounds were selected on the basis of their range in h5-HT_2B_ receptor affinities, and the diversity of the lipophilic and electronic nature of their 4-position substituent. Initially, the compounds were screened at 10,000 nM; those showing agonist action at a concentration below 10,000 nM were then further evaluated by examining their concentration-response relationships (Table 1-B; Figure 1). They displayed reduced efficacy as agonists (i.e., *ca*. 0.7–0.9, excluding **1k** and **1m**; Figure 1) relative to serotonin, and were substantially less potent than 5-HT (EC_50_ = 1.42 nM) with the most potent analog, DOB (**1d**), being about 6-fold less potent than 5-HT (Table 1-B). Two of the compounds, 4-hexyl analogue **1k** and 4-benzyl analogue **1m**, failed to produce an agonist effect at 5-HT_2B_ receptors at the highest concentration examined (10,000 nM), and both behaved as antagonists of the 5-HT-mediated response (Figure 2).

**FIGURE 3:**
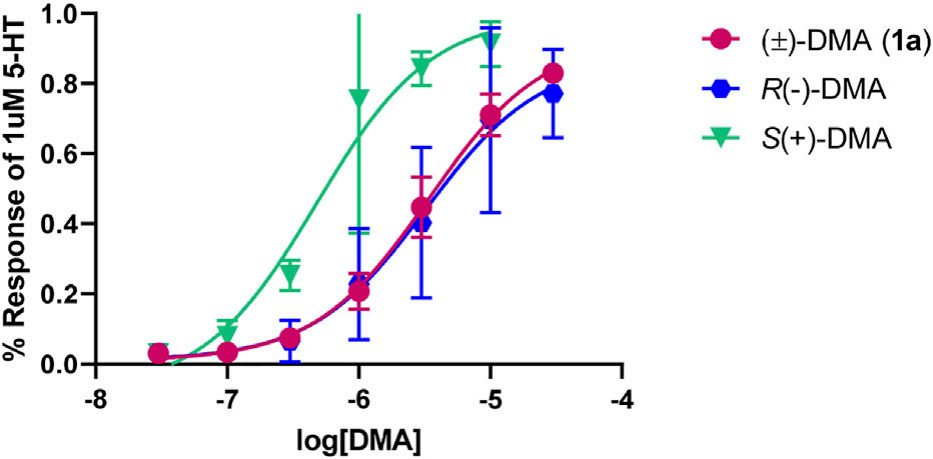
Concentration-response curves for 2,5-DMA (**1a**) and its optical isomers at the 5-HT_2B_ receptor, normalized to 1 µM of the endogenous ligand (5-HT) response.

The individual optical isomers of the parent member of the series, 2,5-DMA (**1a**), were examined at the 5-HT_2B_ receptor; it was found that *S* (+)1a was about six times more potent (pEC_50_ = 6.32 ± 0.15, EC_50_ = 480 nM; 100% 5-HT effect) than its *R* (−) enantiomer (pEC_50_ = 5.49 ± 0.16, EC_50_ = 3,236 nM; 87% 5-HT effect) (Figure 3).

### 3.3 QSAR studies

Using the previously published h5-HT_2A_ receptor binding data from Table 1-A, affinity was found to be correlated with lipophilicity of the 4-position substituent (i.e., π; r = 0.798) alone, and with lipophilicity plus σ_p_ (r = 0.917). Whole molecule Volume (Å^3^), as calculated using SybylX2.1.1 (see Table S1 in SI), was also examined for the compounds in Table 1-A, but volume was found to be inter-correlated with the substituent constant π (r = 0.857). For comparison, rat 5-HT_2_ binding data for this same series of 13 compounds (Table 1-A) also was found to correlate with (π; r = 0.897) alone, and with lipophilicity plus σ_p_ (r = 0.951). A similar QSAR analysis was performed with the h5-HT_2B_ binding data shown in Table 1-A. Affinity was found to be correlated with lipophilicity (i.e., π; r = 0.750) alone, and with lipophilicity plus σ_p_ (r = 0.812). However, the contribution of the σ_p_ term was not statistically significant.

## 4 Discussion

Considerable rat brain homogenate 5-HT_2_ receptor binding data for various phenylisopropylamines **1** and other serotonergic compounds such as, for example, phenylethylamines, tryptamines, β-carbolines, and ketanserin analogs have been published. Once human 5-HT_2A_ receptors were identified and cloned, it was necessary to determine if binding data for agents at the former rat population reflected data from the latter human studies. Indeed, some studies have already used rat 5-HT_2_ binding data for phenylisopropylamines as a surrogate for 5-HT_2A_ receptor 3D-QSAR studies (e.g. Schulze-Alexandru et al., 1999). Despite differences in species (rat *versus* human), radioligands (antagonist [^3^H]ketanserin *versus* agonist [^125^I]DOI), and potential methodological differences, there was no qualitative difference in receptor affinity of the agents examined here. True, this information might not be extrapolated at the current time to non-phenylisopropylamines. However, as might have been expected, on the basis of the use of an agonist radioligand, human 5-HT_2A_ receptor affinities for the examined agents (Table 1-A) were somewhat higher than those for rat 5-HT_2_ receptors. In addition to the antagonist *versus* agonist nature of the radioligands employed, differences in affinity might also reflect species (>90% sequence homology between rat and human). But, here, this does not seem to make a difference for the phenylisopropylamines (i.e., **1** analogs) examined due the affinity correlation shown in Figure S1 (SI). Encouraging also was that QSAR studies, those published earlier with rat 5-HT_2_ binding data (Glennon and Seggel 1989) as well as that presented here with human 5-HT_2A_ receptors, both implicated the lipophilicity and, possibly, the electronic nature of the 4-position substituents as being contributors to receptor affinity; that is, increased affinity is associated with increased lipophilicity and, perhaps, electron withdrawing character.

The lipophilic and electronic character of the 4-position substituents of **1** were also implicated in their binding at h5-HT_2B_ receptors. Furthermore, eight of the 10 compounds in Table 1-A, the exceptions being **1k** and **1m**, behaved as 5-HT_2B_ receptor agonists. Porter et al. (1999) have previously examined the functional action of DOB (**1d**) and DOI (**1e**) (i.e., measurement of intracellular calcium levels in CHO cells expressing human 5-HT_2B_ receptors) and found them to be of similar agonist potency and produced 69% and 65% of the effect of 5-HT, respectively. Also, Huang et al. (2009) have demonstrated that DOI (**1e**) is a potent 5-HT_2B_ receptor agonist in multiple functional assays of receptor activation. However, given the results in Table 1-A and Figure 1, it is evident that functional potency does not appear to be related to 5-HT_2B_ receptor affinity (e.g. compare DOB, **1d**, with its hexyl counterpart **1k** which have nearly identical affinities) and, hence, because affinity is related to the lipophilic and, less significantly, electronic nature of the substituents, the latter measures cannot be taken as reliable predictors of functional potency. Benzyl analog **1m** also failed to produce an effect. A feature that appears to differentiate the demonstrated agonists from the inactive compounds seems to be the “size” of the 4-position substituent; that is, compounds **1k** and **1m** possess the most lipophilic, and largest (volume-wise), of the substituents examined (see Supplementary Table S1; SI). This will require further investigation.

Although lacking agonist action, the hexyl and benzyl analogues (**1k** and **1m**, respectively) bind at 5-HT_2B_ receptors with high affinity; hence, the possibility exists that they might represent antagonists. This was shown to be the case in Figure 2. These same two compounds had also been shown earlier to lack rat 5-HT_2A_ agonist action and behave as antagonists (Seggel et al., 1990; Glennon 2017).

Very few QSAR studies have been conducted on the binding of phenylisopropylamine derivatives at human 5-HT_2B_ receptors. Nistala (2018) examined 22 5-HT_2B_ receptor ligands, that included the compounds in Table 1, using comparative molecular field analysis (CoMFA) and concluded that the lipophilicity of 4-position substituents make a positive contribution to binding, but that substituents on the terminal amine were detrimental. Hajjo et al. (2010) using a very large and diverse data set (and although some phenylisopropylamines were included, analogs of **1** were not) developed useful *in silico* QSAR models for the identification of compounds that might bind at 5-HT_2B_ receptors; useful as they might be, the models were “[unable to] distinguish agonists from antagonists” (Hajjo et al., 2010). The present study found that QSAR studies can aid our understanding of h5-HT_2B_ (and h5-HT_2A_) receptor binding, but are as yet unable to predict 5-HT_2B_ (or 5-HT_2A_) agonist action.

Overall, the present investigation i) demonstrated that the human 5-HT_2A_ receptor affinities for 13 analogs of **1** parallel their rat 5-HT_2_ receptor affinities and that, as a consequence, their interactions might share a common SAR, ii) showed that the human 5-HT_2B_ receptor affinities for these same agents parallel their rat 5-HT_2_ receptor affinities, iii) confirmed that rat 5-HT_2_ receptor affinity is associated with the increased lipophilicity and, perhaps, electron withdrawing nature of the 4-position substituents of **1**, and shows that this relationship also applies to their interactions at human 5-HT_2A_ and 5-HT_2B_ receptors, iv) examined the functional activity of 10 analogs of **1** at human 5-HT_2B_ receptors, and found that 5-HT_2B_ agonist action could not be predicted simply on the basis of receptor affinity or the QSAR studies conducted (i.e., two high-affinity 5-HT_2B_ ligands lacked agonist action in functional assays). On this basis, rat and h5-HT_2A_ receptor binding data might be employed to estimate the affinity, but not the functional activity, of phenylisopropylamines for h5-HT_2B_ receptors. We have previously demonstrated that agonist and antagonist phenylisopropylamines need not share a common SAR and might bind differently at 5-HT_2A_ receptors (Rangisetty et al., 2001); additional studies should now be conducted with phenylisopropylamines that bind at 5-HT_2B_ receptors.

Studies with the phenylisopropylamines fenfluramine and dexfenfluramine (two of the most widely investigated 5-HT_2B_-associated valvulopathogens) have demonstrated that their duration of use was strongly predictive of adverse cardiovascular events (Hopkins et al., 2003; Dahl et al., 2008). Nevertheless, given that certain analogs of **1** have been found on the clandestine market, and shown here to behave as 5-HT_2B_ receptor agonists, it would seem that persistent or chronic use of such agents might lead to cardiovascular complications. This requires further investigation. Recent studies have shown that certain other phenylalkylamines (Kolaczynska et al., 2019; Luethi et al., 2019) including mescaline analogs (Kolaczynska et al., 2021) can activate 5-HT_2B_ receptors. Additional studies with these (and related) agents to identify binding and functional pharmacophores for 5-HT_2B_ action would appear warranted.

## Data Availability

The original contributions presented in the study are included in the article/Supplementary Material, further inquiries can be directed to the corresponding authors.
