# Supplementary material for: Binding and functional structure-activity similarities of 4-substituted 2,5-dimethoxyphenyl isopropylamine analogues at 5-HT2A and 5-HT2B serotonin receptors
Source: Front Pharmacol. 2023 Jan 24;14:1101290. doi: 10.3389/fphar.2023.1101290 (PMC9902381; doi:10.3389/fphar.2023.1101290)
Supplement: Supplementary file 1 [file Table1.DOCX]

Supplementary Material

# Supplementary Tables and Figures

## Supplementary Table

**Table S1.** Lipophilic (π) and electronic (σ_p_) character^a^ of 4-position substituents of **1** analogs shown in Table 1-A, and calculated^b^ whole-molecule volume of analogs **1a-1m** used in correlational studies.

|  |  |  |  |  |  |
| --- | --- | --- | --- | --- | --- |
|  | R |  | π | σ_p_ | Volume (Å^3^) |
| **1a** | H |  | 0 | 0 | 690.29 |
| **1b** | F |  | 0.14 | 0.06 | 700.52 |
| **1c** | Cl |  | 0.71 | 0.23 | 721.46 |
| **1d** | Br |  | 0.86 | 0.23 | 733.82 |
| **1e** | I |  | 1.12 | 0.18 | 748.20 |
| **1f** | OMe |  | -0.02 | -0.27 | 752.38 |
| **1g** | OEt |  | 0.38 | -0.24 | 798.02 |
| **1h** | NO_2_ |  | -0.28 | 0.78 | 748.64 |
| **1i** | CN |  | -0.57 | 0.66 | 736.02 |
| **1j** | nPr |  | 1.55 | -0.13 | 828.22 |
| **1k** | nHex |  | 3.05 | -0.13 | 987.63 |
| **1l** | tBu |  | 1.98 | -0.20 | 850.36 |
| **1m** | Benzyl |  | 2.01 | -0.09 | 950.22 |
| ^a^Values from Hansch, C.; Leo, A.; Unger, S. H.; Kim, K. H.; Nikaitani, D.; Lien, E. J. (1973) Aromatic substituent constants for structure-activity correlations. *J. Med. Chem.* 1973, *16*, 1207-1216.  ^b^Whole-molecule volume was calculated using Sybyl X2.1.1 (i.e., all structures were sketched in Sybyl X2.1.1 and minimized using Tripos force fields with Gasteiger Hϋckel charges and were subjected to 100,000 iterations. The dielectric constant was set at 4 to mimic the environment of the protein. Since the (R)-enantiomer was the eutomer for phenylisopropylamines at 5-HT_2A_ receptors, all structures were sketched with the (R) configuration. Volume (Å3) was calculated using a MultipleVolume algorithm within the Sybyl X2.1.1 (View_Surface and Ribbons_Create_MultipleVolume). | | | | | |

## Supplementary Figures

**Supplementary Figure 1.** Relationship between human 5-HT_2A_ (Nelson et al. 1999) and rat brain (frontal cortex) 5-HT_2_ (Seggel et al. 1990) receptor affinities for analogues **1** (r = 0.942; n = 13).

**Supplementary Figure 2.** Relationship between human 5-HT_2B_ (Nelson et al. 1999) and rat brain (frontal cortex) 5-HT_2_ (Seggel et al. 1990) receptor affinities for analogues **1** (r = 0.916; n = 13).
